# Crises and Turnaround Management: Lessons Learned from Recovery of New Orleans and Tulane University Following Hurricane Katrina

**DOI:** 10.5041/RMMJ.10354

**Published:** 2018-10-04

**Authors:** Marc J. Kahn, Benjamin P. Sachs

**Affiliations:** 1 The Peterman-Prosser Professor and Senior Associate Dean at Tulane University School of Medicine, New Orleans, LA, USA; 2 Department of Pharmacology, Tulane University School of Medicine, New Orleans, LA, USA; 3 The AB Freeman School of Business, Tulane University, New Orleans, LA, USA; 4 Department of Medicine and Office of Admissions & Student Affairs, Tulane University School of Medicine, New Orleans, LA, USA; 5 Visiting Professor of Obstetrics and Gynecology, The Ruth & Bruce Rappaport Faculty of Medicine, Technion–Israel Institute of Technology, Haifa, Israel; 6 Sackler Faculty of Medicine, Tel Aviv University, Tel Aviv, Israel

**Keywords:** Crisis management, education, health-care reform

## Abstract

By their very nature both man-made and natural disasters are unpredictable, and so we recommend that all health-care institutions be prepared. In this paper, the authors describe and make a number of recommendations, regarding the importance of crisis and turnaround management using as a model the New Orleans public health system and Tulane University Medical School post-Hurricane Katrina. Leadership skills, articulation of vision, nimble decision making, and teamwork are all crucial elements of a successful recovery from disaster. The leadership team demonstrated courage, integrity, entrepreneurship, and vision. As a result, it led to a different approach to public health and the introduction of new and innovative medical education and research programs.

## INTRODUCTION

On Monday August 29, 2005, Hurricane Katrina came ashore on the Mississippi Gulf Coast as a category 3 storm (on the Saffir–Simpson Hurricane Scale). The storm stretched across some 645 km and had sustained winds of 160–225 km per hour. The following morning, August 30, the levees, engineered to protect the city of New Orleans, failed. There were an estimated 53 breeches of the levees, flooding over 80% of the city. Approximately 1,836 deaths were directly attributed to the storm, although it is not known how many people subsequently died or suffered because of a lack of health care, stress, or hurricane-related injuries.^1^ The damage to property exceeded US$150 billion, and parts of the Mississippi Gulf coast disappeared. Over 1 million people from Louisiana, Mississippi, and Alabama fled the storm, with the US Census estimating a nearly 50% decrease in New Orleans’s population between 2000 and 2006, the year following the hurricane. Although the storm hit on Monday August 29, 2005, it took until the following Friday for the US federal government to come in force to save the residents of New Orleans.^2^ During these five days the physicians and nurses at Charity Hospital and surrounding health-care facilities in New Orleans had to work under unbelievable conditions, with no electricity or water, in buildings reaching 35 degrees Celsius and 100% humidity. According to the Federal Emergency Management Agency (FEMA), Katrina was “the single most catastrophic natural disaster in US history.”^3^

The first author (M.J.K.) is the Peterman-Prosser Professor and Dean of Students at Tulane Medical School (New Orleans) who experienced Hurricane Katrina as a faculty member and administrator; the second author (B.P.S.) was recruited, in November 2007, as the Senior Vice President, Dean and Doty Distinguished Professor of Tulane Medical School. He had worked at Harvard Medical school for 29 years, and at the time he was recruited he had been the Rosenfield Professor at Harvard Medical School & Harvard School of Public Health (1997–2007), Chair of the Department of Obstetrics, Gynecology, and Reproductive Biology at Beth Israel Deaconess and Harvard Medical School (1989–2007), and the President of the Beth Israel Deaconess Physician Organization (1999–2007).The following are lessons learned from the recovery of Tulane and the New Orleans’s public health system, and, from our perspective, what worked and did not work. These lessons learned are shared because catastrophes can occur in other places and times, and the lessons themselves are generalizable to many other circumstances. Specifically, Israel is at risk of both a major war and natural disasters such as an earthquake with or without a tsunami.

Israel sits on the boundary of two tectonic plates: to the west the African Plate (Golan Heights and Jordan) and to the east the Arabian Plate (Galilee, West Bank, Coastal Plain, Negev, and the Sinai Peninsula). The region has a long history of lethal earthquakes. The historian, Josephus Flavius, reported that an earthquake in 31 BCE killed 30,000 people in the Jordan Valley. In 1927, approximately 500 people died in the “Jericho Earthquake” affecting Jerusalem, Jericho, Ramle, Tiberias, and Nablus, and both the Church of the Holy Sepulcher and the al-Aqsa Mosque experienced major damage. Because of politics and location, Israel’s vulnerability to war and its aftermath goes without saying.

## PRE-KATRINA NEW ORLEANS

Tulane University School of Medicine (TUSOM) was established as the Medical College of Louisiana in 1834. Located in downtown New Orleans, TUSOM is one of the oldest medical schools in the deep south of the United States and was mentioned in the 1910 Flexner Report as one of only a few schools in the southern USA as being educationally sound in its academic programs.^4^ Since its inception, TUSOM has been closely affiliated with Charity Hospital, which was founded in 1736 as a New Orleans hospital for the poor. Prior to Hurricane Katrina, in August 2005, TUSOM had close clinical affiliations with the Veteran’s Administration Hospital New Orleans (VAHNO) and Tulane University Hospital and Clinic (TUHC). In 1996, TUHC was purchased by the for-profit entity Columbia HCA, now known as Hospital Corporation of America (HCA). It is run as a joint venture between Tulane and HCA. Prior to Hurricane Katrina, TUSOM had its challenges: TUSOM was undercapitalized and was threatened with probation by the Liaison Committee on Medical Education (LCME). Several of its graduate medical education (GME) programs were in jeopardy of losing accreditation.

Pre-Katrina, New Orleans had a population approaching half a million people. Over half of the population was African-American. Louisiana had the highest homicide and incarceration rates in the USA and was ranked 49th out of 50 US states in respect to health outcomes.^5^ Upwards of 30% of the New Orleans population lived below the poverty level, the public-school system was failing, and nearly 30% of the population was medically uninsured so that the majority of their care, including primary care, was provided through the Emergency Department at Charity Hospital. Louisiana had the highest cost per capita of federally funded Medicare and the worst health outcomes of any state in the USA.^5^,^6^ This is not surprising, given that so many residents had poor care for most of their lives and that once people became eligible for Medicare at age 65 it was too late to reverse their chronic diseases. To make matters worse, Louisiana had one of the strictest criteria for state and federal health-care assistance (Medicaid). Medicaid is a health-care program that assists low-income families or individuals in paying for doctor visits, hospital stays, long-term medical custodial care costs, and more. Louisiana required a family of four to have an income below $4,500 to qualify for Medicaid and had the provision that adults without children were completely ineligible for financial assistance regardless of income.^7^

## HURRICANE KATRINA

From a public health perspective, five hospitals in New Orleans were destroyed by Hurricane Katrina, including the 550-bed Charity Hospital and the 400-bed Veteran’s Administration Hospital. The TUHC and the Tulane University sustained over US$900 million in damage.^8^ Tulane University declared financial exigency and borrowed US$100 million to stay solvent. The School of Medicine lost one-third of its faculty due to attrition or dismissal. Research facilities, including records and specimens, were destroyed. As a temporary measure, TUSOM moved its educational programs to Houston, Texas, 350 miles from home for the entire 2005–2006 academic year.^9^ Morale was low, post-traumatic stress disorder was rampant, and teaching beds were in short supply.^10^

Following the hurricane and its resultant loss and destruction, the question was often asked: “Why save New Orleans?”^11^ From an economic perspective, the answer was simple. The Port of New Orleans and the Port of South Louisiana in the nearby town of Laplace combine to form the largest port system in the world based on annual tonnage. Additionally, Louisiana ranks in the top three states in the USA for oil and natural gas production, responsible for 30% of total US production, and leads the country in offshore oil production. From a cultural perspective, New Orleans is unique, blending French, Spanish, Creole, Native American, and Anglo roots. As a result, New Orleans has its own cuisine and music that rivals any in the world. In many ways New Orleans represents the best in America: economic opportunity, a unique culture, and a diverse population. It is in many ways the quintessential American city.

## POST-KATRINA RECOVERY

In planning for recovery, we recognized that the faculty, residents, and students who returned and remained in New Orleans after Katrina were people of immense courage who, despite enormous personal challenges, were determined to save New Orleans, its community, and Tulane University. Many if not most professionals had the choice of leaving the city. Yet, they bravely decided to stay and help build a safer and better New Orleans.

The Dean and Senior Vice President of TUSOM (B.P.S.) officially took on this new role in November 2007. However, strategy planning began three months beforehand, with commutes from Boston to New Orleans and Washington DC. Following the storm, Tulane University had hired three consulting firms and had been through a number of strategic planning processes with little to show for the efforts. The new Dean recognized that there would be no tolerance among the faculty for more strategic planning. What was needed was definitive action. A major concern was that unless decisive actions were taken, faculty would leave. During the three months prior to his official start date, the new Dean commuted from Boston, spending a significant amount of time listening, learning, and taking notes about the social, political, economic, and health-care situation in New Orleans, TUHC, and Tulane University. Furthermore, the new Dean built a network of contacts in New Orleans with the help of friends and colleagues, including business and civic leaders from around the country. The Dean recognized that if he were to make a difference in New Orleans, he would need to have strong relationships with business leaders and local community leaders, including religious figures, politicians, the African-American, Vietnamese, and Hispanic Communities, and above all the Federal Government.

In the Dean’s initial message to the Tulane community, he wanted to give a sense of purpose and pride and to encourage entrepreneurship. The key points he made were:

- We will help to build a different health-care system, *not rebuild*.
- The time to act is now. No more consultants or “strategic planning.”
- Turn adversity into opportunity.
- The door to the Dean’s office is always open. All are welcome to come to discuss problems with their recommended solutions.

**Figure 1:**
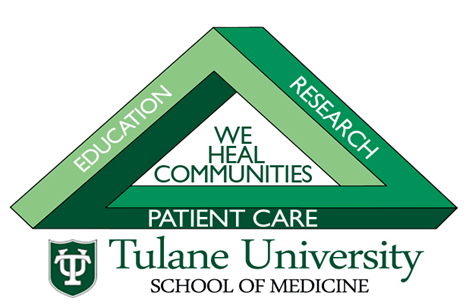
Post-Katrina Logo for Tulane University School of Medicine Emphasizing the Tripartite Mission of Education, Clinical Care, and Research in the Context of Healing the Community of New Orleans.

All medical schools have the tripartite mission of education, research, and clinical care. The Tulane faculty developed a new vision/mission statement in the form of a logo with the motto “We Heal Communities” as the fourth mission (Figure 1). The Dean believed that this message would allow the Tulane community to move forward to take care of patients, to do research, to educate trainees, and to better the city of New Orleans. This new vision provided the rallying cry to recruit students and faculty to the School of Medicine. Faculty and students were drawn to Tulane, motivated to help build a *new* New Orleans. In fact, under the leadership of the new Dean, the faculty numbers grew by over 100%, applications to the School of Medicine increased by 25%, and the average grade point average and Medical College Admissions Test scores of incoming medical students increased.^12^ Healing communities was the vision for the future.

## CLINICAL “REVOLUTION”

Following Katrina, in 2006, Dr Karen DeSalvo (Tulane faculty) started one of the first new primary care clinics with funding from the Government of Qatar. In Boston, community health centers form the basis of primary care services for the underserved; this was in stark contrast to pre-Katrina New Orleans, that depended on the emergency department of Charity Hospital for “primary care.” The Dean saw the tremendous opportunity to rebuild health care in the region using this approach.^13^ To help facilitate Tulane’s mission and to have a role model in the post-Katrina environment, Dr Karen DeSalvo was appointed as a Professor and Vice Dean for Community Affairs and Outreach. By February 2018, Tulane had developed a business plan (known as the Tulane University Plan to Advance the Development of Community-based Health Centers in New Orleans) to demonstrate the economic viability of the neighborhood health center concept. Much to our surprise, civic and political leaders showed little interest in this concept. All they wanted was to reopen Charity Hospital, as this was their reference point. We realized that their only experience was with the old Charity Hospital concept of a hospital for the poor that provided both inpatient and outpatient services in a single inner-city location. They had never seen a network of successful community-based primary care clinics such as the Federally Qualified Health Centers (FQHCs) that exist throughout the USA. The FQHCs are not-for-profit, consumer-directed health-care organizations that provide access to high-quality, affordable, and comprehensive primary and preventive medical, dental, and mental health care.^14^ Thus, FQHCs have a unique mission of ensuring access for underserved, underinsured, and uninsured patients and are provided with federal subsidies for their operations.

The leadership under the Dean decided to change perceptions in 2008 by arranging for a group of civic, community, and religious leaders and the newly appointed Vice Dean to visit Boston to see how these neighborhood health centers functioned. This trip was accompanied by significant media coverage to publicize our notion of a new model of primary health-care in post-Katrina New Orleans. Additionally, having politicians and business leaders witness first-hand the value of neighborhood health centers greatly assisted our achieving local buy-in for this plan. A turning point in our efforts was when a black pastor who was on the Boston trip said that it was the first time that he had ever seen anyone like himself treated with respect in a health-care facility.

At that time, the US Secretary of The Department of Health and Human Services (DHHS) in the President Bush administration was Secretary Michael Leavitt. He was deeply committed to helping New Orleans recover after the storm. His administration, together with the State Government, arranged for a Medicaid waiver where state and federal funds could be used to help fund the growth of primary care clinics. Thus, with funding and the political will, our vision became a reality. By 2010, there were 25 organizations operating in 93 sites providing care for 220,000 people, representing 20% of the population. As further evidence of Tulane’s success in rebuilding the community, in 2010, Tulane University School of Medicine was awarded the Spencer Foreman Award for Outstanding Community Service by the Association of American Medical Colleges. This award singles out institutions that serve as examples of social responsibility through their outreach to their communities.

In September 2010, New Orleans Mayor Mitch Landrieu and the new Secretary of DHHS Kathleen Sebelius (President Obama administration) wrote in an article under the heading “City’s network of clinics a model for national health reform”: “It seems fitting that New Orleans and its people, who have shown the nation how to survive unthinkable tragedy, can now set an example for strengthening the nation’s health care system into the future.”^15^

Looking forward, the state and federal government began to arrange funding for the rebuilding of Charity Hospital under the new name, “University Medical Center.” Ground was broken in April 2011. The US$1.1 billion new hospital opened in August 2015 as a 213,677 sq. meter hospital with 446 acute care beds, 19 operating rooms, and 56 emergency department exam rooms. The new facility was situated 6.4 meters above flood elevation and was constructed with emergency power backup and the ability to withstand the impact of a category 3 major hurricane.^16^ In a similar fashion, the Southeast Louisiana Veterans Health Care System was rebuilt in New Orleans and reopened in the summer of 2017. The new 160,000 sq. meter 440-bed facility was built at a cost of US$1 billion.^17^

## EDUCATION

From an educational perspective, it was clear that conventional strategies were not going to be enough. The two new teaching hospitals were only opened in 2015 and 2017. As a result, the patients and beds that were needed to support the undergraduate and graduate medical education enterprise were scarce in New Orleans. The first innovation was the establishment of a clinical campus in Baton Rouge, 75 miles from New Orleans. Following the storm, New Orleans was no longer the most populated city in Louisiana due to the exodus of its residents. Baton Rouge, the state capital, was the new population center. Creating a new program located in the state capital allowed for a thematic approach to undergraduate medical education. The campus was created along the theme of leadership: *Lead*, *Educate*, *Advocate*, *Discover*. In addition to typical clinical rotations (internal medicine, surgery, obstetrics and gynecology, etc.) specific opportunities to explore community development, entrepreneurship, involvement in the political process, organized medicine, and practical experiences in the Governor’s Office and Department of Health were created.

In addition, recognizing the high cost of training a physician and the amount of time necessary, the School of Medicine created an accelerated undergraduate-to-MD program that uniquely included a mandatory year of public service with AmeriCorps VISTA. Consistent with the mission of healing communities, allowing highly motivated undergraduates the path to an MD in six years plus a year of service was mission-consistent and was expected to lead to bright young physicians with an emotional attachment to the city.

Building on the reputation of the city as a culinary capital and recognizing the poor state of health of the New Orleans residents, the school, under the leadership of Dr Timothy Harlan (Associate Dean for Clinical Services) developed The Goldring Center for Culinary Medicine at Tulane University. This is the first dedicated teaching kitchen to be implemented at a medical school. The center provides hands-on training for medical students through culinary medicine classes in the form of electives and seminars as well as continuing education for the health-care and foodservice industries. Of note, as of 2018, the nutrition curriculum developed by the center is being used in approximately one-third of all medical schools in the USA.^18^

**Figure 2:**
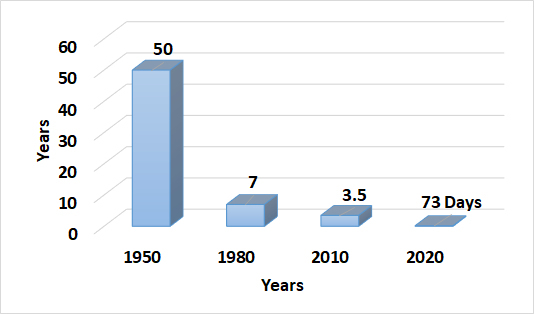
Estimated Doubling Time of Medical Knowledge by Year Students starting medical school in 2010 will master 6% of the knowledge available in 2020. Adapted from Denson, 2010.^17^

Finally, under the direction of Dr Kevin Krane, Vice Dean for Academic Affairs, new methods of teaching were put into the curriculum. It is estimated that the doubling time of medical knowledge has gone from 50 years in 1950 to 3.5 years in 2010. By 2020, the doubling time is estimated to be only 73 days (Figure 2).^19^ As a result, medical students of the future need to be “adaptive learners,” able to apply existent knowledge to new situations.^20^ Recognizing this concept, new modalities of instruction as alternatives to classroom lectures were developed. These included Just in Time Teaching, Team-based Learning, and other flipped classroom techniques that provided instruction using pedagogy more consistent with adult learning theory. As an immediate result, student pass rates on national board exams were higher than the national average for the first time in many years.

The rapid expansion of medical knowledge, as described, will fundamentally change the role of a physician in ways that are hard to predict today. One possible solution, advanced by Tulane, was the development of unique dual degree programs. The physician of the future needs to be more than just a good diagnostician. As such, the School of Medicine constructed a four-year combined MD/MBA program. The school had a five-year program for several years prior to Katrina, but recognizing the value of time a four-year program with an emphasis on health care was developed. This program was one of only a few of its kind in the USA and was used as a recruitment tool by the School of Medicine to attract students who were not only academically talented but also interested in management and being part of the creation of a new infrastructure for health-care delivery.

## RESEARCH

As with their clinical and education programs, Tulane University School of Medicine was forced to readdress the focus of their research programs following Hurricane Katrina. As a result of the storm, the school lost significant research assets due to failure of refrigeration and power, as well as losing research faculty due to layoffs and resignations. The Dean decided to expand research by focusing on research programs that were interdisciplinary, involving investigators not only from the School of Medicine but also from the schools of science and engineering, law, and business. Through funding available from the National Institutes of Health as part of the post-recession (2008) recovery stimulus, the school was able to build modular laboratory space that was flexible and open. Rather than a scientist working alone in an individual laboratory, this new space encouraged groups of scientists, across disciplines, to work together in a collaborative fashion. Synergies in research interests and geographical proximity were hoped to create novel research programs designed to address the needs of both the New Orleans and Tulane community. Tulane also focused recruitment of new faculty and Department Chairs to individuals whose funding and research interests dovetailed with Tulane’s strengths in research that included infectious diseases, heart disease, and cancer biology. Through these efforts, Tulane realized its best years in securing extramural NIH funding since inception, with steady growth of 10%–20% in NIH research dollars per year following the new vision.

## LESSONS LEARNED

Sometimes it takes a disaster to remove silos and create a new vision. Based on our experience, the following are our key recommendations:

1. Understand the culture: For an outsider coming in to help the recovery after a major disaster, it is essential to understand, appreciate, and show respect for the local culture. Build a broad coalition of local leaders: No matter how wonderful the plan/vision for recovery, without obtaining local “buy-in” and support, change is very unlikely.
2. Build a strong leadership team: The road to recovery will be long and hard, and recovery will never be achieved by a micro-managing single leader. It is important, from the outset, to build a management team and to delegate decision making but at the same time to hold the team responsible. As part of this approach, consider empowering entrepreneurship and encourage managers to take risks.
3. Have a recovery plan, but be prepared to adapt based on local conditions: The frequent approach following a disaster is to hire consultants, organize retreats, and develop strategic plans. We believe this is the wrong approach, because it is too time-consuming. In a crisis, there is a significant risk of the loss of key personnel and for the community to lose confidence. A vibrant vision will give people a sense of hope. In preparing for the position, the new Dean interviewed a wide group including the Tulane community and civic leaders. As a result, when B.P.S. presented his vision, the community felt they had input.
4. Vision alone is insufficient: When the vision includes a radical new approach, words alone cannot build support. Our goal was to build a network of neighborhood clinics to replace the use of Charity Hospital’s emergency room for primary care. To build broad support for this approach, we took key community leaders to Boston to show them the value of neighborhood health clinics. We learned that explanation is no substitute for first-hand experience.
5. Manage post-traumatic stress disorder (PTSD): Following disaster, expect widespread psychological problems among adults and children that have survived the crisis. It is vital for the leadership to show compassion and recognize the needs of everyone involved. This will require the careful allocation of scarce resources.
6. Lead by example.

## LONG-TERM CHALLENGES

Despite the many successes in the transformation of Tulane University School of Medicine and the health-care system in New Orleans following Katrina, several long-term challenges remained. The first was maintaining a system to care for the poor and indigent population with limited financial resources. The Affordable Care Act (ACA) was enacted in the USA in March of 2010 and provided for expanded coverage for the poor through expansion of state and federal funding through Medicaid. However, state challenges to the legality of provisions of the ACA resulted in a Supreme Court majority decision that allowed individual states to choose to not participate in federally supported Medicaid expansion. Louisiana was such a state that did not expand Medicaid, with an estimated loss of over US$1 billion of federal subsidy and an estimated human cost of over 1,000 lost lives per year.^21^ It was not until January 2016 that a new governor, John Bel Edwards, agreed to Medicaid expansion in Louisiana.

A second challenge, “Katrina fatigue,” required a constant reinforcement of the vision and mission to a community that was still experiencing the post-traumatic effects of the storm.

As health-care facilities were rebuilt, maintaining bed occupancy remained a challenge as the city was over-bedded prior to the storm. This resulted in intense competition for insured patients. To maintain market share, hospitals made plans for consolidation and reorganization that increased feelings of uncertainty among health-care providers and institutions. In fact, the replacement hospital for the old Charity Hospital, in order to remain financially solvent, sought to compete with other hospitals in New Orleans for insured patients, resulting in mission drift.^22^ Additionally, due to political forces the LEAD program was discontinued in Baton Rouge, and Tulane decreased its medical school class size in 2015.

Finally, external factors such as increased risk of major hurricanes due to global warming, the loss of Louisiana wetlands that provide some protection from hurricane tidal surges, and rising sea levels continue to leave the city of New Orleans vulnerable to significant weather-related damage. Although the levees that protect the city were rebuilt, they are far from perfect. In addition to the risk of flooding, Louisiana continues to face annual budget deficits such that the New York Times has called Louisiana a “Failed State.”^23^ Louisiana state law allows for reducing a budget shortfall only by reducing expenditures in two areas: education or health care.

## CONCLUSIONS

Although it is impossible to completely plan for disasters, it is clear that political instability, the risk of war, climate change, and geography make disruption a potential reality for any health-care system. Articulation of vision, nimble decision making, maintenance of mission, and focus on results are all essential features of successful recovery from disaster. Following Hurricane Katrina, Tulane University School of Medicine and the New Orleans public health system were able to rebuild as well as reform education, research, and clinical care. Remaining challenges include budgetary and funding issues and the possibility of another Katrina-like storm. Despite these challenges, the reforms have proven, in most cases, to be sustainable and better adapted to the challenges facing the health-care profession.

## Abbreviations

FEMA: Federal Emergency Management Administration
FQHC: Federally Qualified Health Center
NIH: National Institutes of Health
TUHC: Tulane University Hospital and Clinic
TUSOM: Tulane University School of Medicine
